# Supplementary material for: The More Natural the Window, the Healthier the Isolated People—A Pathway Analysis in Xi’an, China, during the COVID-19 Pandemic
Source: Int J Environ Res Public Health. 2022 Aug 17;19(16):10165. doi: 10.3390/ijerph191610165 (PMC9408338; doi:10.3390/ijerph191610165)
Supplement: Supplementary file 1 [file ijerph-19-10165-s001.zip › ijerph-1809060-supplementary.pdf]

Supplemental material

The More Natural the Window, the Healthier the Isolated People – A Pathway Analysis in Xi'an, China During the COVID-19 Pandemic.

Wangqin Bi, Xinyi Jiang, Huijun Li, Yingyi Cheng, Xingxing Jia, Yuheng Mao, Bing Zhao

TABLE OF CONTENTS

Figure S1. The final SEM model 2 demonstrates standardized effects between variables.

Table S1. Effects of green view and mediators on anxiety in the structural equation model.

Figure S2. Structural equation model linking green view to depression.

Figure S3. Structural equation model linking green view to anxiety.

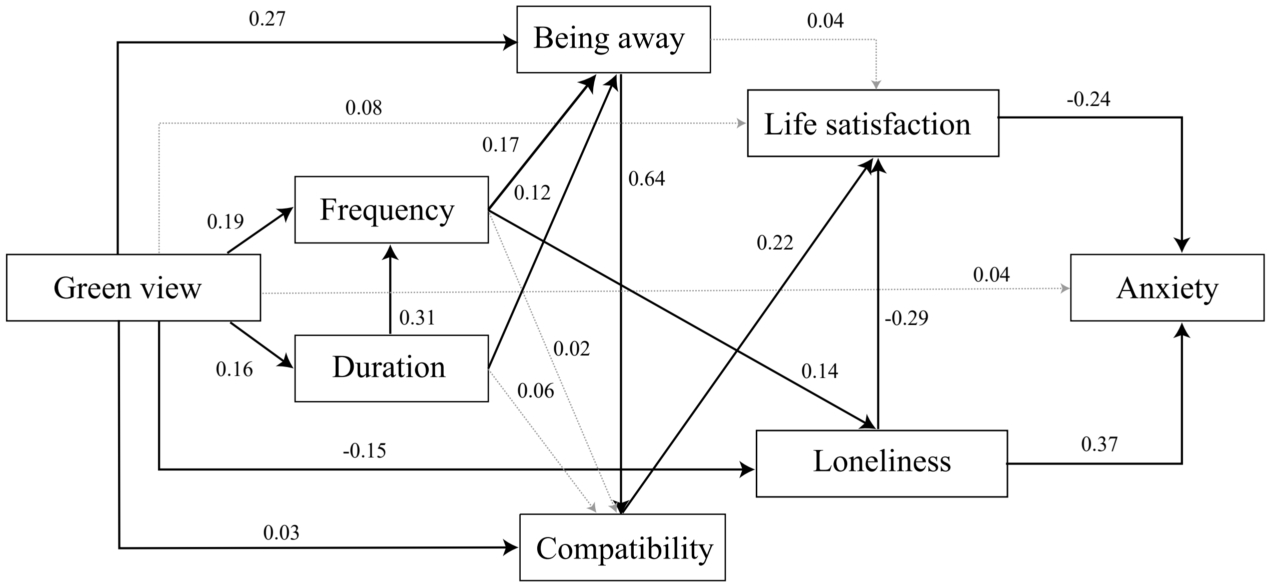

Figure S1. The final SEM model 2 demonstrates standardized effects between variables.

Note: The black solid lines indicate $p < 0.001$, and the gray dotted lines indicate $p > 0.05$.

Parameter	SE	Estimate	Bias-corrected percentile method		percentile method	
			Lower	Upper	Lower	Upper
**Total effects**						
Green view	0.030	−0.026	−0.084	0.032	−0.084	0.031
Duration	0.004	0.004	−0.003	0.012	−0.003	0.012
Frequency	0.012	0.030	0.008	0.057	0.007	0.056
Bing away	0.005	−0.110	−0.022	−0.004	−0.021	−0.004
Compatibility	0.005	−0.015	−0.028	−0.006	−0.026	−0.006
Loneliness	0.016	0.146	0.116	0.178	0.116	0.178
Life satisfaction	0.015	−0.063	−0.093	−0.034	−0.093	−0.034
**Indirect effects**						
Green view→ Compatibility→ Life satisfaction→ Anxiety	0.001	−0.003	−0.007	−0.001	−0.006	−0.001
Green view→ Loneliness→ Life satisfaction→ Anxiety	0.002	−0.006	−0.011	−0.002	−0.011	−0.002
Green view→ Loneliness→ Anxiety	0.010	−0.030	−0.054	−0.012	−0.051	−0.011
Green view→ Loneliness→ Anxiety	0.002	0.005	0.002	0.011	0.002	0.010

Table S1. Effects of green view and mediators on anxiety in the structural equation model.

Note: Coefficients are unstandardized linear regression coefficients. SE=bootstrap standard errors.

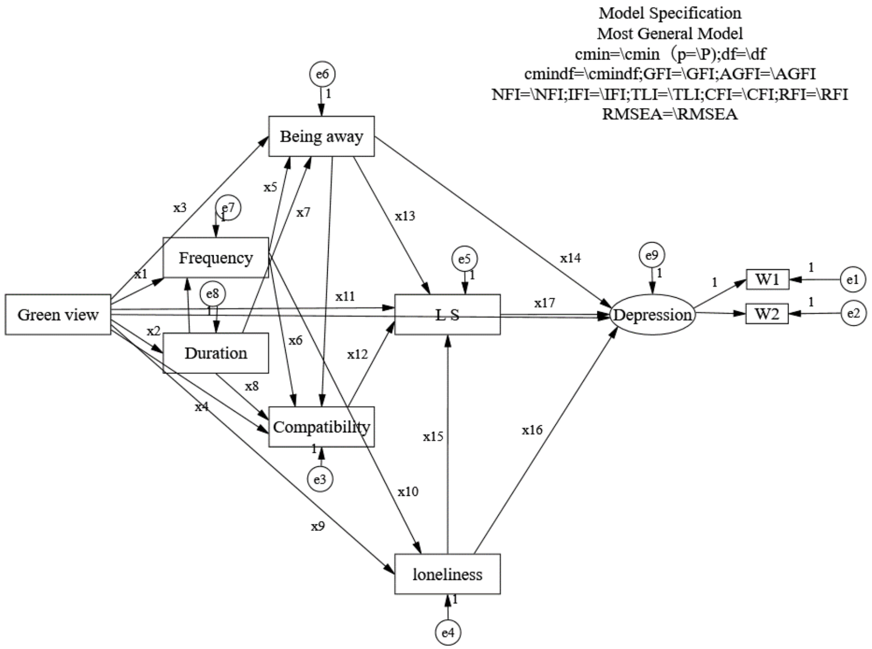

Figure S2. Structural equation model linking green view to depression.

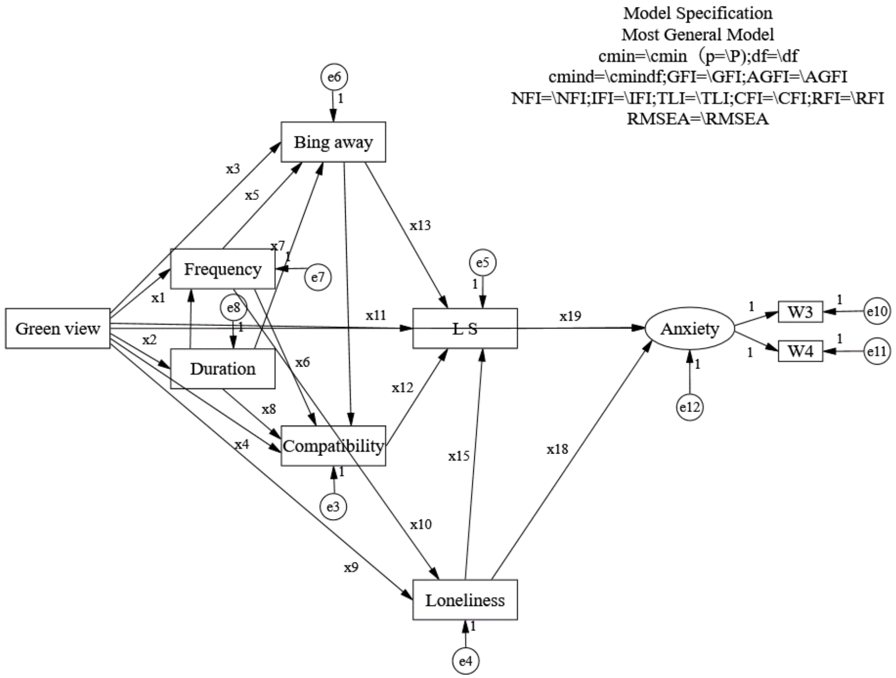

Figure S3. Structural equation model linking green view to anxiety.
